# Anti-aging protein SIRT1: A role in cervical cancer?

**DOI:** 10.18632/aging.100031

**Published:** 2009-03-06

**Authors:** Christopher L. Brooks, Wei Gu

**Affiliations:** Institute for Cancer Genetics, and Department of Pathology, College of Physicians & Surgeons, Columbia University, New York, NY 10032, USA

**Keywords:** p53, SIRT1, activation, transcription, small molecule, cell growth arrest, stability, cellular senescence, metabolism, aging

The sirtuin protein SIRT1 is a well studied and highly complex NAD^+^ dependent class III histone deacetylase that has seemingly diverse functions in metabolism, aging and cancer. SIRT1 deacetylates several downstream effector proteins including KU70, NBS1, the FOXO transcription factor family, and p53, several of which are in response to DNA damage events occurring within the cell [1]. Regulation of these factors by SIRT1 has indicated its direct role in cell growth and cellular senescence, and recent advances in understanding the physiologic nature of SIRT1 in these processes have posited both growth-promoting and grow-inhibiting effects of the protein.

The transformative properties of the Human Papilloma Virus (HPV) were originally exploited well over 40 years ago as tools for elicudating mechanisms of cell growth control and cancerous transformation [2]. Indeed, the tumor suppressor p53 was first shown to be regulated by the ubiquitin-proteosome pathway by its association with the cellular ubiquitin ligase E6-AP, a protein upregulated by HPV E6 protein [3]. High risk HPV genotypes (HPV16, -18, -31,  and -33) are now known as direct, causative agents of cervical cancer and the E6 and E7 proteins are widely known to induce the degradation and downregulation of p53 and pRb, respectively, as a mechanism for commendeering the growth of the host cell [4]. Until now, however, it has been unclear what other, if any, cellular factors HPV E6 and E7 target for immortalizing cells. In this issue of *Aging*, Allison et al. show that HPV E7 upregulates cellular SIRT1 and induces global histone modifications within the cell as an additional mechanism for promoting cell survival and inhibiting apoptosis.

 Significant research intensity has been placed on understanding the role SIRT1 has in cellular transformation and cancer with studies showing both growth-promoting and grow-inhibiting effects of the protein. It has been suggested that SIRT1 does not fit the classical definition of a *bona fide* tumor suppressor as it does not induce cell growth arrest when overexpressed in cell culture and has no documented cases of deleterious point mutations in any type of human tumor [5]. The growth suppressing observations *in vivo*, therefore, could be attributed to the genetic context of p53 [6]. In cells retaining wildtype p53, SIRT1 regulates and maintains p53 in an inactive state by deacetylating the transcription factor and promoting cellular senescence over apoptosis. However, in cells lacking p53, such as p53 null tumor cells, SIRT1 may lose repression from tumor suppressors such as p53 and HIC1 allowing for the overexpression of SIRT1. Several p53 negative tumor cell lines overexpress SIRT1 and become highly dependent on the protein as shown by apoptosis induction after these cells are treated with SIRT1 siRNA [7,8]. The numerous p53-independent functions that SIRT1 is involved with, such as metabolic pathways and histone modification, may help these cells escape an apoptotic fate and promote tumorigenesis.

In this issue of *Aging*, Allison, Jiang, and Milner have shown for the first time that a DNA tumor virus targets the anti-aging protein SIRT1 and exploits protein function as a mechanism for promoting uncontrolled cell growth. It is well known that specific genotypes of HPV (HPV 16 and HPV 18) are causative agents of cervical cancer with nearly 500,000 new diagnoses each year [9]. Previously, it was thought that the major mechanisms that the virus uses to circumvent controlled cell growth are through the selective degradation of p53 and pRb, two critically important growth control tumor suppressors. HPV E6 recruits the cellular ubiquitin ligase E6-AP to ubiquitinate and degrade p53. By maintaining low p53 protein levels, the cell is unable to mount a stress response to the invading virus. However, previous observations indicated that a p53 response in cervical cancer cells by siRNA depletion of HPV E6 led to cell growth arrest but not apoptosis, indicating that another cellular pathway was being overcome by HPV to block apoptosis [10]. Allison et al. show that HPV E7 selectively upregulates SIRT1 in SiHa cervical cancer cells. Upregulation of SIRT1 provides two downstream effects: prevention of apoptosis, as seen in other tumor cell lines that overexpress SIRT1, and deacetylation of p53, thereby inhibiting its ability as a transcription factor to induce apoptosis.

HPV E6 and E7 proteins are coded for by a bicistronic mRNA, which makes it difficult to assess the individual contributions of HPV E6 and E7 on cellular transformation. In this study, it was found that HPV E6 and E7 could be selectively silenced by siRNA in HPV 16-positive SiHa cells allowing for functional assessment of each protein separately. Interestingly, selective silencing of HPV E6 was not sufficient for apoptosis induction, whereas silencing of HPV E7 induced apoptosis despite HPV E6-mediated p53 suppression. These data suggest that selective targeting of both p53 and SIRT1 by HPV is required to block apoptosis and cell growth arrest as part of the transformation process. HPV E7 was also shown to upregulate global histone H3 S10 phosphorylation, presumably through upregulation or stabilization of survivin. Global chromatin modifications during transformation are thought to lead to chromatin instability [11] and the previously unknown direct and/or indirect mechanisms of HPV E7 may have a significant effect on cervical cell tumorigenic transition. Other downstream effectors of SIRT1, such as FOXO3, may also play a role in this transition if SIRT1 is constitutively expressed, since FOXO3 deacetylation reduces FOXO-mediated apoptosis [12-14].

The malignant transformation of cervical cells by HPV from a latent state is not well understood. The virus exists as an episomal chromosome for an indeterminate amount of time within a cell until cellular transformation occurs and the virus integrates into the host genome [15]. Stable integration of the DNA also enhances HPV E6 and E7 expression, which allows the virus to combat p53 and SIRT1, respectively [16]. It is unclear at what point of the viral lifecycle that the HPV E7-mediated induction of SIRT1 is important, though Allison et al. show that exogenous expression of HPV E7 in primary keratinocytes causes significant and rapid upregulation of SIRT1 within 48 hours, whereas siRNA reduction of HPV E7 in SiHa cells caused reduction of SIRT1 and induced apoptosis. Together, these data suggest a role of overexpressed SIRT1 in HPV-infected cervical cells for preventing apoptosis, contributing to cellular transformation, and in conjunction with p53 downregulation, allowing unregulated cell growth to occur.

HPV E6 has received intense focus in small molecule drug discovery for disrupting E6-mediated E6-AP protein induction and subsequent p53 degradation. However, these data would suggest that focus on HPV E7 is warranted as a more suitable target since siRNA-mediated reduction of HPV E7 in SiHa cells induces apoptosis. Conversely, reduction of HPV E6 leads to p53-mediated cell growth arrest, but a full apoptotic response requires inhibition of both viral proteins. These data could have profound implications on the focus of drug discovery for HPV therapeutics and suggests a re-analysis of current target strategies. Though p53 is a major factor for controlling cell growth and is targeted by HPV E6, the current study has shown that HPV E7 and the upregulation of cellular SIRT1 has significant effects on the tumorigenic growth of HPV infected cervical cancer cells. Importantly, downregulation of HPV E7 is sufficient for inducing apoptosis in these cells, indicating that selective targeting of this protein may be an effective strategy for reversing cervical cancer tumor growth.

The current study also provides insight into general regulation of SIRT1 and specific roles it may have in tumorigenesis and cancer. The downstream effect of SIRT1 overexpression and p53 degradation mediated by HPV E6 and E7 is reminiscent of other tumor types that lack functional p53 and overexpress SIRT1, a condition seen in several non-cervical cancer cell lines [8]. Under these conditions, cells become dependent on SIRT1 overexpression, presumably due to other p53-independent mechanisms that allow the cell to continue to grow in an uncontrolled manner. These cells are highly dependent on overexpressed SIRT1 and undergo apoptosis upon siRNA knockdown. Similarly, the HPV transformed SiHa cells undergo apoptosis when SIRT1 levels are reduced by HPV E7 siRNA knockdown. SIRT1 may act as an anti-apoptotic factor in transformed cells or cancer cell lines that lack a functional p53. The differential effects of SIRT1 could therefore be specific to the genetic background of the cell or tumor in question and may not fit into the classical definitions of an oncogene or tumor suppessor.

Identification of SIRT1 as a downstream target of HPV E7 has broad implications in understanding HPV and cellular transformation. HPV E6 has long been known for targeting p53, but HPV E7 has now been shown to be involved with p53 regulation as well through targeting SIRT1. As small DNA viruses were originally used as tools to understand and elucidate cancerous growth, they have once again provided invaluable insight into cellular factors involved in cellular transformation. This is not only important for developing novel therapeutics for treating HPV infection, but is crucial for understanding the physiologic function of SIRT1 in cancer and aging. Is SIRT1 a critical component in the viral latency to malignancy transition? Is SIRT1 an important anti-apoptotic factor in transformed cells that have lost functional p53? Is overexpression of SIRT1 a general mechanism for maintaining global de-acetylation in tumor cells? Answering these questions will undoubtedly provide new insight into the molecular mechanisms of cellular transformation and tumorigenesis for both cervical cancer and other tumor cell types overexpressing SIRT1.
